# Estimating the impact of administration of dewormers on smallholder chickens in Odisha State, India

**DOI:** 10.3382/ps/pey526

**Published:** 2018-11-27

**Authors:** Paul R Bessell, Ranjit Dash, Sanjay Prasad, Lamyaa Al-Riyami, Neil Gammon, Kristin Stuke, Roy Woolley, Miftahul Islam Barbaruah

**Affiliations:** 1 Epi Interventions, 32 Bell Place, Edinburgh, EH3 5HT, UK; 2 Gir Odisha Foundation, Kadei, Badachana, Jajpur, Odisha 754296, India; 3 Vet Helpline India Pvt Ltd., H.No 31/32 Milanpur (Near Masjid No.1), Chandmari, Guwahati 781021, Assam, India; 4 GALVmed, Doherty Building, Pentlands Science Park, Bush Loan, Penicuik, Edinburgh, EH26 0PZ, UK; 5 GALVmed, Galana Plaza, 4th Floor, Wing C, Suite B, Galana Road, Kilimani, P.O. Box 52773-00100, Nairobi, Kenya

**Keywords:** dewormer, chicken, India, weight, smallholder

## Abstract

Helminth infections, in particular infections with nematodes are highly prevalent and an impediment to the productivity of chickens in smallholder settings. Infections can be easily and cheaply treated using dewormers. We present an empirical framework for estimating the impact of administration of locally available dewormers on chicken weight in a smallholder setting in Odisha State of India.

We recruited 1,040 chickens aged between 40 and 70 d from 168 households in 13 village groups in Odisha. Chickens were randomly assigned to treatment with a dewormer (fenbendazole), or non-treatment. Each chicken was tagged with 2 legbands and weighed, then followed up after 28 and 56 d and reweighed. To account for the local variations in exposure and for variations between flocks, the data were analyzed in a multilevel mixed model with flock within village as nested random effects.

After 56 d, the modeled results showed that all chickens had gained a mean of 288.3 g but heavier chickens at the baseline gained more weight than lighter chickens. In addition to this, the treated chickens had gained an additional mean of 90.55 g relative to non-treated chickens (*P* < 0.001).

In this setting, we have demonstrated that administration of dewormers has a clear beneficial impact on chicken weight, but it also indicates that other management practices can have a substantial impact on chicken weight.

## INTRODUCTION

Smallholder farming is vital to agricultural production in India with chickens a major component. Nematode infections in chickens are very common in smallholder chickens, with prevalence ranging from 35 to 100% with a large number of nematode species typically identified, including *Ascaradia galli, Hetarakis gallinarum*, and *Capillaria spp.* (Permin et al., 1997; Poulsen et al., 2000; Mungube et al., 2008; Nnadi and George, 2010; Kumar et al., 2015). Infections by other types of helminths—cestodes and trematodes are less frequently reported. Helminth infections have a clear impact on the development and productivity of the infected species, primarily affecting the weight gain (Sargison et al., 2017). There are a number of off-the-shelf dewormers that are typically administered en masse without obtaining a diagnosis but only a small number of studies have sought to estimate the impact that dewormers have on weight gain of smallholder chickens (Phiri et al., 2007; Chota et al., 2010; Katoch et al., 2012).

The objective of this study was to evaluate the impact of the administration of a locally available dewormer on the weights of smallholder chickens. The study must control for differences in exposures, genetics, and feeding regime, many of which cluster at the level of the household and the village. To account for this, we designed a randomized controlled trial in which treatment with the dewormer was randomized at the level of the individual chicken and within each flock chickens are randomly assigned to treatment or to non-treatment.

## MATERIALS AND METHODS

### Study Hypothesis

- H_0_ (Null hypothesis). Treating chickens with a dewormer has no statistically significant effect on weight gain over a 56-d period.
- H_1_ (Alternate hypothesis). Treating chickens with a dewormer has a statistically significant effect on weight gain over a 56-d period.

### Study Design

The households in the study villages were surveyed to identify those that had chickens in the age range 40 to 70 d to select growers. Households were selected from the survey until the sample size for that village was achieved. Households that were selected for enrolment were asked to return to a central point with the chickens where they were informed of the purpose of the study and asked to sign an informed consent form (IC) that had been translated into the local language. The IC informed the farmer of the purpose and structure of the study and the terms of the financial incentive. If a household declined to sign the IC then another suitable household was enlisted. Details of the enrolled household and its management practices were recorded, and subsequently each chicken was taken in turn and:

1. The chicken was weighed.
2. A coin was tossed to assign the chicken to treatment or non-treatment.
3. If treatment was assigned then the chicken was treated orally with a quantity of dewormer appropriate for the chicken's weight.
4. The chicken was tagged using 2 legbands bearing a code in the format Flock #/Chicken # and the data collected on smartphones using ODK Collect (Hartung et al., 2010).
5. After 28 and 56 d, the chickens were reweighed and the data recorded on ODK Collect.
6. Following the final survey, a small incentive of 200INR = 3USD was paid for all tagged chickens that were present at the final survey. Dewormer was offered for all chickens that were not treated at baseline.

All animal handling was conducted by trained personnel to standards consistent with IACUC guidelines to minimize discomfort to the animal. Animals were monitored for any adverse health effects.

### Treatment and Equipment

The locally available dewormer that was selected was fenbendazole 2.5% W/V (Karnataka Antibiotics Pharmaceuticals Ltd. Bengaluru, India) administered orally at 10 mg/kg body weight (0.4 mL/kg). Farmers were informed of the 6 d withdrawal period for chicken meat. The chickens were weighed using table top scales (Sansui Electronics (P) Ltd, Gultekdi, India). Dewormers were administered directly to the mouth of the chicken using a syringe.

### Study Area and Timing

The project was implemented in the districts of Dhenkanal, Jajpur, Kendrapara, and Cuttack in the state of Odisha in India (described in Bessell et al., 2018). From 12 blocks, a total of 17 villages were sampled. The baseline survey was conducted in December 2016, which was shortly after the wet season when the worm burden is likely to be greatest, with the villages accessible and a period with no locally observed religious festivals that may have been a cause to slaughter chickens.

### Sample Size

The required sample size was 1,040 chickens from a minimum of 13 villages. This was based on a simulation model with a village level prevalence of 20% (beta (10, 40)). Within each village, 20 flocks were fitted with a mean size of 4 birds (poisson (4)), the infection status of each bird was sampled from a Bernoulli distribution with the probability set as the sampled village level prevalence. Individual birds were then assigned to the treatment or non-treatment group from a Bernoulli (0.5) distribution. Infected untreated birds grew at a mean rate of 13.7 g/d (Katoch et al., 2012), treated and non-infected birds grew at a mean rate of 18 g/d (both sampled from a log-normal distribution). This framework was simulated with 10,000 iterations for a sample range of 5 to 20 villages, and at each iteration, *t*-test was performed between the treatment and non-treatment groups. The sample size was the first number of villages at which the *t*-test was significant at *P* < 0.05 for at least 90% of iterations (statistical power of 90%).

### Statistical Modeling

Data were analyzed as a linear mixed model with the final weight of the chicken as the dependent variable and starting weight, age, sex, and treatment status of the chicken the explanatory variables. To control for heterogeneities in exposures, local parasite resistance and for flock level variations in genetics, husbandry, and nutrition, a mixed model framework was used in which the flock and village of the chicken were included as random effects in a nested structure. To find the most parsimonious plausible model, we fit 6 separate models of the final weight (*y*) of the chickens:

$\left( {model{\rm{\ }}1} \right){\rm{\ }}y \approx a + {b_1}\left( {start{\rm{\ }}weight} \right) + {\rm{\ }}{b_2}\left( {treatment} \right) + {\rm{\ }}\epsilon$
$\left( {model{\rm{\ }}2} \right){\rm{\ }}y \approx a + {b_1}\left( {start{\rm{\ }}weight} \right) + {\rm{\ }}{b_2}\left( {age{\rm{\ }}band} \right) + {b_3}\left( {treatment} \right) + {\rm{\ }}\epsilon$
$\left( {model{\rm{\ }}3} \right){\rm{\ }}y \approx a + {b_1}\left( {start{\rm{\ }}weight} \right) + {\rm{\ }}{b_2}\left( {sex} \right) + {\rm{\ }}{b_3}\left( {treatment} \right) + {\rm{\ }}\epsilon$
$\left( {model{\rm{\ }}4} \right){\rm{\ }}y \approx a + {b_1}\left( {start{\rm{\ }}weight} \right) + {\rm{\ }}{b_2}\left( {age{\rm{\ }}band} \right) + {b_3}\left( {sex} \right) + {\rm{\ }}{b_4}\left( {treatment} \right) + {\rm{\ }}\epsilon$
$\left( {model{\rm{\ }}5} \right){\rm{\ }}y \approx a + {b_1}\left( {start{\rm{\ }}weight} \right) + {\rm{\ }}{b_2}\left( {treatment} \right) + {b_3}\left( {start{\rm{\ }}weight{\rm{\ }}x{\rm{\ }}treatment} \right) + {\rm{\ }}\epsilon$
$\left( {model{\rm{\ }}6} \right){\rm{\ }}y \approx a + {b_1}\left( {start{\rm{\ }}weight} \right) + {b_3}\left( {start{\rm{\ }}weigh{t^2}} \right) + {b_3}\left( {treatment} \right) + {\rm{\ }}\epsilon$

Where *a* is the intercept that corresponds to the baseline change in weight among the study chickens over the study and *b_n_* the fitted estimate for each explanatory variable, $\epsilon$ is the error term describing the variance due to the nested random effects of flock in village.

The models 1 to 6 were compared using the Bayesian information criteria (BIC)—looking for the lowest BIC as well as comparing the significance from the analysis of variance of the models relative to model 1. The model was fitted in the R statistical environment (R Core Team, 2017) using the lme4 package (Bates et al., 2015).

## RESULTS

### Baseline Data

The sample size of 1,040 chickens was enrolled from 168 households in 17 villages that formed 12 village groups with some of the villages geographically indistinguishable from others in the same group. The chickens were enrolled from 168 households (mean 6.19 chickens/household; minimum 2, maximum 17), all households provided supplementary feed and night shelter for their chickens. Knowledge of dewormers was minimal with only 2 respondents aware of dewormers and none having previously used dewormers.

### Descriptive Statistics

Extensive descriptive analysis of this study is presented in a non-peer-reviewed report (Bessell, 2017). There was loss to follow-up of 15% (primarily due to death of chickens), and this was slightly greater among the non-treated group, the treated group accounting for 55.4% of chickens after 56 d compared to 54.7% at baseline (Table 1).

**Table 1.** Summary of chickens enrolled and treatment status.

	Total	Not treated	Treated
Number of chickens enrolled			
Baseline	1040	471 (45.3%)	569 (54.7%)
28 d	969 (93.2%)	438 (45.2%)	531 (54.8%)
56 d	883 (84.9%)	394 (44.6%)	489 (55.4%)
Mean weight in g (sd; range) at baseline	373.0 (203.5; 101–1128)	391.1 (208.0; 102–1128)	358.0 (198.6; 101–940)
Sex			
Female	758 (72.9%)	341 (32.7%)	417 (40.1%)
Male	282 (27.1%)	130 (12.5%)	152 (14.6%)
Mean age in days at baseline	52.6	52.9	52.3

SD = Standard deviation

There was little difference between treated and non-treated chickens after 28 d, but after 56 d, the treated chickens had gained 15.8% more weight than the non-treated chickens (Table 2).

**Table 2.** The differences in chicken weight at the different time points.

	28 d		56 d	
	Not treated	Treated	Not treated	Treated
Mean difference (g)	219.8	225.9	444.9	515.1
SD difference (g)	94.5	102.0	126.1	131.8
Range (g)	31–715	34–938	110–850	183–1015

SD = Standard deviation

### Impact of Treatment on Weight Change

Of the 6 models evaluated, those including age and sex (models 3, 2, and 4) were not significantly different to the basic model (model 1) and had a greater BIC (Table 3). The model including an interaction (model 5) was significantly better, but had a higher BIC, whilst the model including a quadratic term (model 6) describing the non-linear relationship with starting weight was also significantly different and had the lowest BIC and was considered the best model fit (Table 3).

**Figure 1.**
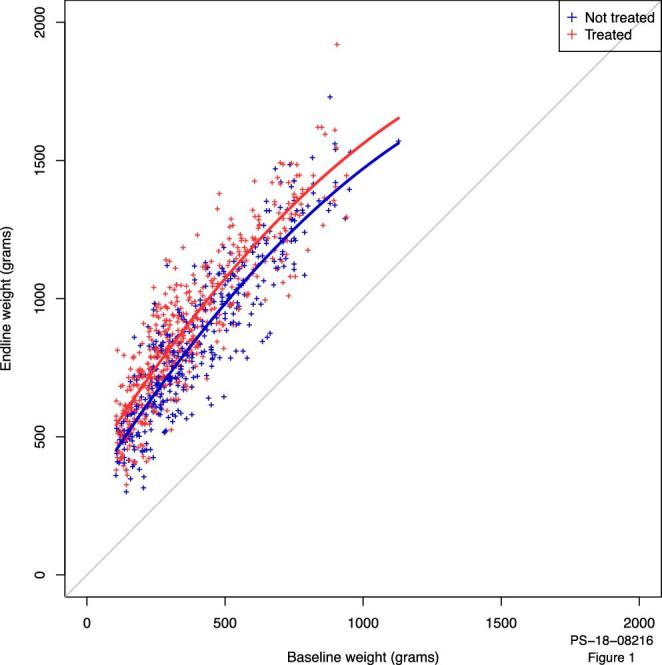
Scatterplot of starting weights vs endline weights (after 56 d). The lines represent the final fitted model based on the parameters in Table 4.

**Table 3.** Results of model comparison from anova analysis.

Model	Degrees of freedom	BIC	Chisq	*P*-value
Model 1 (Ref)	6	10,607	–	–
Model 3	7	10,613	0.26	0.61
Model 5	7	10,610	3.21	<0.001
Model 6	7	10,588	22.63	<0.001
Model 2	8	10,617	0	1
Model 4	9	10,624	0.26	0.610

After the starting weight had been taken into account, treatment had an additional effect of 90.55 g over non-treatment which at baseline gained the intercept of 288.3 g (Table 4). There was also a significant effect of starting weight, with heavier chickens at baseline gaining more weight than lighter chickens (*P* < 0.001 when compared to equality = 1) but this was tempered by small effect of the non-linear relationship with starting weight so the effect of starting weight drops off with increasing weight (Table 4, Figure 1). Accordingly, a 500 g untreated bird at baseline will gain 584 g and a 1 kg bird 880 g.

**Table 4.** Summary outputs of the final linear mixed model of the impact on chicken weight.

	Estimate	Standard error	*T*-value	*P*-value
Intercept	288.3	25.22	11.44	<0.001
Starting weight (g)	1.592	0.078	20.35	<0.001
(Starting weight (kg))^2^	$-$0.410	0.080	$-$5.14	<0.001
Treatment				
Not treated	–	–	–	–
Treated	90.55	5.924	15.29	<0.001

## DISCUSSION

In this study, none of the smallholder farmers had previously used dewormers. In spite of this, the overall effect of deworming was significant but not large, the chickens gained a baseline of 288.3 g and treatment contributed a further 90.55 g. It is notable that each gram of weight at baseline contributes a further 1.592 g at endline (tempered by a small non-linear effect). This effect of starting weight could reflect the growth curves of chickens or could suggest an effect of management practice among this study population that some birds were larger at baseline is due to them being given more or better food.

The magnitude of the effect of treatment with dewormers may reflect the study design that selected younger chickens to ensure measurable weight change and to assist in the retention of chickens. However, among growers the infection rate may be lower than adults due to the lower lifetime exposure, consequently the observed impact of dewormers may also be lower. Furthermore, if infections that were present at the baseline were immature then that would explain the relatively small impact observed after 28 d. Little variation in the residuals at the village level was seen in model diagnostics indicating that there is little anthelminthic resistance at the village level. Furthermore, since dewormers were never used resistance is unlikely, but further field work would be required to conclusively establish this.

Analysis of the prevalence of locally circulating parasites would inform analyses of these results to tailor the intervention to the locally circulating parasite species, but we were unable to find any published reports of local parasite species. In this study, we did not identify the locally circulating parasite species because this would not be conducted by the farmers. Furthermore, in this setting it would be very challenging to collect a truly representative longitudinal sample of circulating parasites without modifying the study design and integrity of the study. Hence, we are only considering the impacts of the treatments rather than attempting to diagnose the infections.
